# Violent events, ward climate and ideas for violence prevention among nurses in psychiatric wards: a focus group study

**DOI:** 10.1186/s13033-016-0059-5

**Published:** 2016-04-05

**Authors:** Tella Lantta, Minna Anttila, Raija Kontio, Clive E. Adams, Maritta Välimäki

**Affiliations:** Department of Nursing Science, University of Turku, Hoitotieteen laitos, TURUN YLIOPISTO, 20014 Turku, Finland; Helsinki University and Hospital District of Helsinki and Uusimaa, Helsinki University Central Hospital, Helsinki, Finland; Division of Psychiatry, Institute of Mental Health, University of Nottingham, Nottingham, UK; School of Nursing, The Hong Kong Polytechnic University, Hong Kong, China; Turku University Hospital, Turku, Finland

**Keywords:** Focus groups, Patient assault, Psychiatric Hospitals, Psychiatric nursing, Qualitative research, Violence, Violence prevention, Workplace violence

## Abstract

**Background:**

Patient violence against nurses in their work environments is a widespread global concern, particularly in the field of mental health care. A high prevalence of violent events impacts the well-being of nurses and may also impair overall ward climate. However, it has been proposed that nurses’ use limited techniques to prevent patient violence, and, therefore, more comprehensive methods for dealing with patient violence are needed. There is still restricted understanding of the ward climate during the occurrence of a violent event as well as how these incidents could be more effectively prevented. This study aimed to explore nurses’ experiences of violent events in psychiatric wards, give insight into ward climates and examine suggestions for violence prevention.

**Methods:**

This study employed a descriptive, exploratory design including focus groups (n = 5) and open-ended questions. The participants were registered and enrolled nurses (n = 22) working on three closed psychiatric in-patient wards in one Finnish hospital district. Focus groups were tape-recorded, transcribed and analyzed with inductive content analysis.

**Results:**

Nurses’ experiences of violent events included a variety of warning signs and high-risk situations which helped them to predict forthcoming violence. Patient-instigated violent events were described as complicated situations involving both nurses and patients. When the wards were overloaded with work or emotions, or if nurses had become cynical from dealing with such events, well-being of nurses was impaired and nursing care was complicated. Suggestions for violence prevention were identified, and included, for example, more skilled interaction between nurses and patients and an increase in contact between nurses and patients on the ward.

**Conclusions:**

This study revealed the complexity of violent events on psychiatric wards as well as the implications of these events on clinical practice development and training, administration and policy. A routine process is needed through which nurses’ experiences and ideas concerning prevention of violent events are acknowledged.

## Background

Patient violence against health care personnel at work is a widespread global concern, particularly in the fields of mental health care [1] and, more specifically, psychiatric nursing [2]. For example, in the USA, about 40 % of all nurses have been exposed to physical violence and 70 % to violence of a non-physical type [3]. A recent systematic review and meta-analysis revealed that nearly 20 % of patients admitted to acute psychiatric wards may behave violently [4]. For health care organizations and staff, violent events involving patients can bring about medical expenses, potential legal expenditure [2], sick leave and a high turnover rate [5]. For patients, these events can mean longer periods of stay, higher medication use and more readmissions [6]. Violent events may also have an impact on nurses’ well-being in the form of, for example, post-traumatic symptoms [7], fear [8], work-related stress [9], anxiety, blame [10], and the feeling of being insulted [11]. A high prevalence of violent events may also impair the overall ward climate [12] and thereby erode the quality of patient care [13]. Preliminary evidence shows that a less than ideal patient environment, for example overcrowding in psychiatric hospitals, may increase the risk of violence directed at staff [14].

Violence has been defined as physically or psychologically harmful human aggression involving the threat or use of force [15]. Violence may be subjected to various targets, for example, toward nurses [16], other patients [1] or objects [17]. Nursing staff often perceive patients’ acute state of mental illness as the main cause of violent events [18, 19]. This is, however, not in keeping with the views of patients, which emphasize interpersonal problems as being a cause for the majority of violent events [20, 21].

It has been proposed that the techniques used by nurses to prevent patient violence are limited [22] and that more comprehensive methods for dealing with patient violence are needed [19, 23]. More recently, less restrictive and coercive measures [24] and safer ward environments have indeed been developed [25, 26]. Previous studies have shown that a positive working climate is beneficial in preventing incidents of patient aggression on wards [27, 28]. Patients themselves are also more satisfied with the nature of the treatment on wards where the number of violent events is low [29]. A positive environment is thought to be achieved through continuing education and managerial support [25], better medical management of patients [21], and/or improved handling of interpersonal problems with more flexibility regarding limit-setting [21]. There is still insufficient understanding regarding the nature of ward climates (the social and therapeutic atmosphere [30]) during violent events and how violence prevention could be used more effectively form the viewpoint of nurses.

This study explores nurses’ descriptions of violent events that have taken place on psychiatric wards, the ward climate, and suggestions for preventive activities. The research questions for this study are: (1) how do nurses describe violent events on psychiatric wards, (2) how do nurses describe the ward climate during violent events, and (3) what are the suggestions of nurses for how violence prevention could be more effective.

The study is a part of a larger Finnish “Safer working management”- project, which aims to develop new methods for caring for violent patients on psychiatric wards and promoting nurses’ well-being at work.

## Methods

### The design of the study

This study followed a descriptive, exploratory design using focus groups. As information regarding this topic is scarce, an explorative approach was appropriate for this study, which aims to reveal a deeper understanding about this particular phenomenon [31]. Also, focus groups are deemed to be a suitable technique used for examining topics perceived to be sensitive [32]; they have previously been used in studies that examine the experiences of health care staff regarding violent events [33].

### The setting

The study was conducted in one hospital district in southern Finland. Within Finnish health care services, it has been estimated that 46 % of nurses who work on psychiatric wards have suffered from work-related violence [34]. The specific district selected for this study was done so because, according to statistics from the Finnish National Institute for Health and Welfare, it had an average level of seclusion and restraint use, and involuntary admissions, within the realm of psychiatric care in Finland. For example, in this district there were 143 people per 100,000 inhabitants who had experienced ‘involuntary hospital days’ in 2011, while the average number in Finland is 155 [35]. Moreover, 29 patients had been secluded and 16 physically restrained in this district (the average in Finland was 30 and 14 per 100,000 inhabitants in 2011) [35].

Out of the 20 wards that showed possible interested in participating (there was a total of 30 adult in-patient wards in the district), three in-patient psychiatric wards in three different hospital organizations within this district participated in the study. These three wards were chosen due to the high number of violent events on those wards, the frequent amount of coercive methods being used, and the fact that no other development projects were going on the wards at during the course of this study. Each care unit admits patients needing special treatment in closed wards: (1) an acute admissions ward (15 beds; the most common diagnosis group F29, ICD-10 [36]), (2) an acute forensics ward (18 beds; the most common diagnosis group F20, ICD-10 [36]), and (3) a ward specializing in patients with violent behavior (16 beds¸ the most common diagnosis group F29, ICD-10 [36]).

### Recruitment

Participants in this study were nurses working on the target wards during the data collection period (August 27–September 4, 2012). A purposive sampling method was used to select participants likely to be able to provide relevant information [37]. Predefined inclusion criteria stated that participants be registered or licensed clinical nurses, have a permanent or long-term position (over 3 months), be aged 18 or over, have sufficient command of the Finnish language and be willing to participate in the focus groups. Staff members were excluded in cases where a nurse did not meet patients on daily basis (e.g. nurses on night shift, clinical directors) or if they worked outside of the nursing profession (e.g. physiotherapists, social workers, physicians, psychologists, secretaries).

The nurse manager on each ward acted as a contact person for the study. She/he recruited staff members to participate and ensured that all staff members were contacted. The nurse managers were first informed by the researchers about the study—its purpose, methods and recruitment process [38]. The preliminary plan was to include 4–6 participants in each focus group. Potential participants were contacted by a nurse manager who provided oral and written information about the study. The nurse manager also informed these nurses that they could attend focus groups during their working hours. If a nurse had a day off, they could have participated and received the hours spent in the focus group back as working hours, although this opportunity was not exploited. Nurses willing to participate gave written consent. Five focus groups were formed (range of participants 3–7).

### Focus group questions and the data collection

The content of the focus groups centered on nurses’ experiences of violent events on psychiatric wards. The questions were guided by main themes chosen to provide information on the topic under study: descriptions of violent events, ward climate, and suggestions of how violence prevention could be more effective. Questions were open-ended [39], allowing nurses to describe their experiences, attitudes and suggestions related to the theme, to generate their own questions and pursue their own priorities [40]. An example of the types of questions asked is, “How have the violent events you have described affected the climate of the ward?”

The focus groups were run by two female university researchers (members of the research team: TL, MA). Prior to each focus group meeting, the interviewers introduced themselves to participating nurses, explained the study again both orally and with written information, and reminded them of the voluntary nature of their participation. The participants had opportunities to ask questions. To ensure a relaxed atmosphere, focus group met in peaceful environments at hospital facilities, and refreshments were provided [40].

The focus group meetings were conducted by two people: one had the main responsibility of guiding the conversation of the group and one was responsible for tape-recording, taking written notes and assuring that all relevant themes were discussed. In one focus group, only one interviewer was used due to the other interviewer having a previous working relationship with some of the participants. The quality of the focus group meetings was assured by using trained and experienced interviewers. One interviewer has PhD degree and had previously conducted several research interviews. The other is a MNSc and a registered nurse with experience working on psychiatric wards. Repeat focus group meetings were not carried out.

Out of 61 eligible participants, over one-third (n = 22, 36 %) agreed to participate. Each focus group session lasted 80–110 min.

### Data analysis

Inductive content analysis was used to analyze focus group data [41]. The analysis process was divided into six phases.

- First, taped focus groups were transcribed [40] word for word by one author (TL) (4 focus groups) and a professional secretary (1 focus group). The text included altogether 134 pages (line spacing 1).
- Second, an overall picture was formed from the raw data by reading these transcriptions carefully; the aim was to become familiar with the original expressions that came out in the focus groups [42].
- Third, the transcribed text was read through again, keeping in mind the study questions under investigation and highlighting and making preliminary notes relating to the text at the same time. By doing this, ‘meaning units’ were formulated. A meaning unit can be words, phrases or paragraphs that include aspects related to each other [42].
- Fourth, meaning units related to the research questions were transferred to a separate MS Word document—and partly grouped during transfer. All highlighted meaning units were then condensed to codes [42]. Codes were considered to be more abstract and condensed labels for meaning units [42]. Each code can be reflective of several meaning units. In most cases the labels of codes came directly from the raw data [41].
- In the fifth phase of the analysis, all codes were then grouped into categories based on how different codes are related [41].
- Finally, sub-categories and categories were formed. Some of the sub-categories or main categories, or their definitions, started to emerge even when transcribing the focus group meetings. However, this final phase involved combining and organizing grouped codes into sub-categories and further, to categories [41].

### Validity

The validity of the study was enhanced throughout the study process. The participants represented three different psychiatric wards to increase the credibility for the focus group data [42]. The focus groups were audiotaped along with concurrent written notes in case of machine failure [43]. The focus groups were transcribed verbatim to provide a detailed account [42]. Participants were offered the opportunity to see the transcript for commenting, although no one availed themselves of this. However, the preliminary results of the study were presented to nurses for commenting. Participant feedback confirmed that the results reflected ward reality.

The data analysis was inductive-avoiding preconceived categories [42]. Original expressions of the focus groups were used to add credibility. All five authors have evaluated the categorization and the results of the analysis. Validity was further enhanced by a clear and detailed description of the data collection and analysis [42], thus increasing transparency. The study was reported using consolidated criteria for reporting qualitative research (COREQ) [44].

### Ethics, consent and permissions

An ethical assessment of the study was carried out by the Ethical Committee of the Hospital District (270/13/03/03/2012) and the Ethical Committee of the University (13/2012) which complies with the Code of Ethics of the World Medical Association (Declaration of Helsinki [45]). Permission to undertake the study was obtained from the chief of psychiatry [46].

Oral and written information was offered to each participant and written informed consent was obtained from the nurses before participation [38]. Anonymity of individual participants has been ensured by representing the results with ID codes; participants cannot be identified from written data or study reports. The data was stored in a locked room to which only named researchers of the study group have access.

## Results

### Description of the participants

In total, 22 nurses participated in the focus groups (6 men, 16 women). At the time, nine worked on the acute admissions ward, six on the acute forensics ward, and seven on the treatment and rehabilitation ward, which is specialized in treating patients with violent behavior. To ensure the privacy of the participants, no further demographic data were gathered [47].

### Description of violent events on psychiatric wards

Nurses’ descriptions of violent events were categorized into three main categories:

i. signs of violence;
ii. targets of violence; and
iii. responsive action in violent events.

#### Signs of violence

This category includes the sub-categories of patient-related warning signs and high-risk situations. Patient-related warning signs were, for example, general verbal provocation, e.g. shouting or tone in the voice, gestures, special type of movement (unrest, coming too close) or facial expressions. The nurses explained that patients’ signs are easier to interpret when the nurses know the patient. One nurse described patient-related warning signs the following (the number in brackets identifies the respondent in the data):

*They can be like blinking eyes more often or preening hair* — *(ID 5)*

High-risk situations were related to restrictions—for example, when patients were not allowed to exit the ward, or have visitors or telephone calls. Other examples of restrictions that could lead to violent reactions include giving a patient medication against his or her will or informing a patient about their enforced hospital stay. In these situations, something was denied, which provoked the patient to violent events.

*If one has to inform a patient about things that they will not like, it can be predicted that it might provoke violent behavior* — *(ID 1)*

#### Targets of violence

This category includes the sub-categories of violence towards nurses, violence towards other patients, and violence towards objects. Violence towards nurses was, in most cases, verbal intimidation, such as calling names or criticism. The nurses also experienced physical violence against them. For example, nurses have been bitten, kicked, slapped, pushed and burned with cigarettes. Sometimes, nurses received death threats concerning themselves, relatives or even pets.

— *“I have had all kinds of, I mean threats that ‘I’m going to kill you’. That I will be killed, all my relatives, and even my cat.”* — *(ID 17)*

Violence towards other patients was generally described as being in the form of extortion-related threats. Nurses reported how patients lend each other goods (e.g. cigarettes), the repayment of which causes difficulties and arguments. Occasionally, there are fights between patients and, in rare cases, even attempted manslaughter.

*Between the patients there is more verbal aggression, but there have also been some situations of struggle. (ID 7)*

In addition, targets of violence were described as violence towards objects. This manifested itself in the breaking of windows and the destroying of furniture, doors, taps or entire rooms.

— *an awful pounding was heard, we knew that soon there would be an alarm so we went there, and a patient was trying to break down the window with a chair*— *(ID 4)*

#### Responsive action in violent events

This category includes the sub-categories of patient actions, nurses’ actions and actions of other patients. The actions of the patients were described as various types of attempts at solving. The patient may settle down just by seeing the number of nurses or by having a conversation with a nurse. They may also fight back or continue to make violent attempts. Nurses explained that patients sometimes try to block themselves off from the nurses by using objects such as chairs. When nurses use physical restraints to manage behavior, some patients use all their power to get released. At times, they may manage to escape from the event.

*There have been numerous times when I have seen a patient count how many [staff] were there and do nothing, until when there were less staff* − *which makes the situation ready. (ID 17)*

Nurses’ actions were described as attempts to solve a violent event by avoiding extra damage to people and property. Usually, a nurse’s immediate response is to attempt to take the control of the event by alarming other staff and seeking additional help. Taking care of other patients in violent events is an essential part of a nurse’s response, as well as staying calm him- or herself. Nurses described how they try to use less restrictive interventions and avoid using patient seclusion rooms. For example, they may give the patient the opportunity to withdraw from the situation, allow him/her to go into his/her own room and stay there for a specific time period, go to the seclusion room with open doors, or take oral medication. Nurses also described using aggression management techniques (mainly physical restraints), which they believed to be safer for both the staff and patients. Another option, as described by the nurses, is to try to have a calming conversation with the patient. This method involves intervening in patient violence without hurting anyone. In addition, it was explained that coercive measures are used when less restrictive interventions fail. These include forced medication, seclusion, mechanical restrains and physical restraints. Nurses described seclusion as an especially useful intervention, not used unnecessarily, and they criticized the pressure they are under to use this method less.

*We also offer a kind of opportunity where one goes to the seclusion room with open doors, to have a chance to be alone in peace. Some find being alone to be privilege, there is no one else provoking or whatever. One can go there on your own as well as freely leave. (ID 7)*

During violent events, the actions of other patients often involves them allying with the one behaving violently. Other patients may join in with the violent patient either before the event occurs or when nurses try to intervene. Nurses mentioned that some patients try to get attention just when there is a chaotic event on the ward, e.g. by wanting to make a phone call. Some patients are eager to help the nurses in these events. Still, the most common behavior from the other patients is just to disappear.

— *among the patients, there might have been some hero patient, who tries to protect the nurses from an aggressive rager, e.g. by scooping a nurse into safety in the nurses’ office. (ID 2)*

### Descriptions of ward climate during violent events

The descriptions nurses gave of the ward climate during violent events were categorized into three main categories: overloaded with heavy workload, overloaded with emotions and inducing cynicism.

#### Overloaded with heavy workload

This was typified by frequent and long-term violent events. When violent patients are secluded, the workload increases, affecting several nurses, complicating nursing care and causing a stressful work atmosphere. Nurses are told that they do not have time to take care of other patients, which can then lead to new kinds of unpredictable events.

— *long*-*lasting seclusion, which takes time and attention and energy from several nurses, and if there is less time for other patients, then you notice a kind of general anxiety which occurs* — *(ID 1)*

#### Overloaded with emotions

Ward climate during violent events are loaded with different kind of emotions. Being ‘overloaded’ was said to impair well-being at work, quality of care and also have a negative impact on nurses’ free time. Nurses described anticipatory tension—just waiting for the next episode to come. The events cause tension, also described as a “healthy fear”, and the need to always be alert on wards with violent patients. Nurses preferred the expression, “being alert”, instead of “fear” and were somewhat reluctant to consider their emotions to be fear. Anticipatory tension or fear related to ward climate is something that cannot become visible to a violent patient. Furthermore, nurses emphasized that constant fear would make working impossible.

— *you are afraid, if someone sees your fear, the situation will be actually lost, you must just stay cool. (ID 8)*

— *one cannot work if is afraid all the time. (ID 1)*

#### Inducing cynicism

the occurrence of frequent violent events and an ongoing hostile environment can result in a ward climate that induces cynicism among nurses. Working in that kind of climate can, over a period of time, change a person, to the extent that even physical violence towards nurses may eventually be considered unexceptional. During the focus groups, nurses needed to consider what violence actually is, but, in one example, they expressed that verbal violence is something that they are so used to that they do not always recognize or report it; it was somehow perceived as part of their work. Nurses learn to tolerate “heavy handling” on psychiatric wards where violent events are commonplace. An example of a ward climate’s impact on a nurse’s well-being is illustrated below.

*Somehow, one has learned to tolerate such heavy handling and one may easily start to respond in the same way. If someone is already using violence, the threshold for another to use it tends to become lower. Somebody’s life might derail because of a violent situation* — *(ID 2)*

### Suggestions of how violence prevention could be more effective

The study participants had many suggestions for how violence prevention could be more effective. These suggestions fell into four main categories: in-service training, competent interaction, presence of nurses and security improvements. The nurses expressed that high-quality in-service training for the whole staff would make treatment policies more coherent, whereas security improvements to the physical structure of the ward would, for one, make observing patients easier. However, nurses felt that they could do more as professionals. They felt that competent interaction is one way to prevent violent events from escalating, and that even just being present for patients would make a difference (see Table 1).

**Table 1:** Nurses suggestions how violence prevention could be more effective

Category	Detailed suggestions	Example
In-service training		
To make treatment policies more coherent Should be more high-quality, concrete and offer new knowledge	Information: early warning signs, prediction of violence, new drugs and violence-related subcultures More in-service training for the all staff - especially de-escalation technique training	*To be able to practice together*—*the one who does and what, I suppose everyone knows broadly what to do, but what is their location at the situation, it demands some co*-*practice (ID 6)*
Competent interaction		
Staff-to-patient and staff-to-staff, e.g. between a nurse and a physician Interaction problems with a physician may endanger patient care: e.g. insufficient medication and lack of information provided to a patient	Courage to ask straight whether a patient has violence-related thoughts Consequences of violent behavior should be discussed clearly with the patient The importance of leadership and clear instructions clarification of nurses’ work in violent events A consensus among the staff how to deliver treatment Adequate, stable workforce: when staff knows each other well, interaction is easier	*It’s terribly hard for the patients, too* — *when there is no such line and they don’t have time to get used to anybody* — *it increases the risk that something might happen because such a stabile situation is aimed for in which everything is very consistent and everybody knows how to proceed, what they can and cannot do, it is always such a thing that holds the thing together (ID 17)*
Presence of nurses		
Patients being themselves, if ward climate tense and frustrated and can lead to violence	Familiar nurses provide safety for the patients Time to be present for the patients and a named nurse with primary responsibility to take charge if there are signs of violent behavior Only one of the nursing staff speaks to the patient in case of violent event	*That the more we can be there, so called present and displayed on the ward, so that way we can make the situations more calm (ID 17)*
Security improvement		
Lack of privacy and overcrowded wards Unsupervised places, e.g. smoking rooms, balconies Dysfunctional computers: if the files are unreachable, critical information may not be reported to next shift A patient or a visitor may smuggle drugs or bring weapons into wards	Reduction of beds, increase of single rooms for patients Avoidance of certain one-to-one situations, e.g. being twosome in kitchen with the patient Ensuring functioning electronic equipment for efficient reporting Compliance of security instructions: e.g. locked places should be locked When in doubt, permission to check patients’ bags even against patients’ will Drug detection dog on wards if needed Surveillance cameras and metal detectors on ward exits	*Neglecting such direct safety instructions, for example, locked places are left open, e.g. in the kitchen* -*knife drawers might be left unlocked, I think it is such a security deficit here, as well as dangerous chemicals, such detergents, might be unsupervised that one has a change to drink them for intoxication purposes (ID 3)*

## Discussion

This study explored nurses’ descriptions of violent events on psychiatric wards, ward climate, and ideas for preventive activities. The study demonstrated that the experiences of Finnish nurses working in psychiatric in-patient wards are very similar to those in various other countries and settings.

We found that nurses try to predict violent events by interpreting patients’ signs and triggers. This result supports earlier findings in which nurses have described predicting the risk of violence by observing patients’ warning signs [48] with unstructured methods of appraisal [49]. On the other hand, previous studies have shown that nurses may blame a patient’s mental illness [19, 21, 23] or problems in their interpersonal communication [18, 19, 50] as a main cause of violent events. We found it to be less straightforward—that a real complexity exists in the violent events, including interactions between several parties and modes of action. Nurses may rely on their empirical knowledge [48], which may limit structured decision-making in demanding situations [51]. Omérov et al. [52] have implicated that nurses may misinterpret patients’ signals in relation to their intentions. Therefore, it can be assumed that nurses still need more knowledge and training on how to interpret patient warning signs related to violent events in order to support their decision-making in demanding situations.

Nurses’ actions in violent events were described in various ways. Nurses’ described trying to use less restrictive interventions to manage patient violence, as has been illustrated in many other studies: therapeutic interaction with the patient [11, 22, 52], by offering oral medication [19, 22, 23], and giving the opportunity to withdraw from the situation [52]. However, as was also stated in a few other studies [19, 23], a need for coercive measures, especially seclusion, was highlighted. Nurses described intervention generally in line with international recommendations about the management of patient violence [53]. However, the needs and preferences of an individual patient in both prevention and management of violent events were hardly discussed by the nurses. Nurses may act based on given organizational instructions and customs, regardless of a patient’s individual needs [54]. Based on our results, more widely integrating individual risk management plans [55] or joint crisis planning [56] into psychiatric in-patient care practices might be reasonable and could lead to more person-centered care of violent patients [53].

Our results do support previous studies showing the negative effects of violent events on ward climate [12, 57]. We found ward climates are considered to be overloaded. Nurses were somewhat reluctant to describe their feelings as ‘fear’, favoring references to stress and being constantly alert. This is somewhat different to previous results, where fear related to violent events had been reported more openly by nurses [8, 10]. Some nurses in our study described psychiatric care in a cynical manner, where nurses did not always recognize violence because they had become so used to it. This phenomenon has already been described in psychiatric nursing as being emotionally “hardened-up” [58], being institutionalized [59] or getting hurt being seen “as a part of nurse’s job” [60]. The situation hinted at here could seem alarming—situations on psychiatric wards can suddenly turn violent and the causes have gone unnoticed [58]. Nurses should always be on the lookout for these damaging events [61] and eager to avoid the negative feedback loop, which impairs morale [58, 62].

Participating nurses developed ideas on how to prevent violent events on psychiatric wards, but these ideas were very traditional. They named the need for safer working environments, as reported in several previous studies [23, 25, 63], as well as the need for continuing education [25] and competent interaction skills [20, 21]. This shows that what nurses in earlier studies felt was lacking is still considered insufficiently dealt with, despite the practice of vocational and continuing education and the updating of physical ward environments.

## Limitations

There were several limitations in this study. First, the choice of using Nurse Managers as contact people for recruiting participants may have led to selective sampling. Recruiting participants for sensitive research is always a challenge, however, and maximizing personal contact in the process may have a positive impact on finding the possible participants who are most willing and motivated to share their intimate ideas with researchers [64]. Second, the study sample consisted of nurses working with each other. This may have had a positive, encouraging impact on the focus groups, but may have also hindered some participants to freely share ideas [43]. Third, concerning the data analysis, approaching the text always includes some degree of interpretation influenced by the authors’ occupational histories [42]. To some extent, this was unavoidable, as research experience from psychiatric care might also have provided better possibilities of understanding the data. The validity of findings was increased by having the results evaluated by all authors, and by using the interviewees’ own words and original expressions for the names of categories as much as possible.

## Conclusion

This study revealed how complex violent events in psychiatric wards are, including multiple participants and modes of action. The study showed a need for a routine way of acknowledging nurses experiences and their ideas concerning the prevention of violent events.

This study offers import considerations for practice development, administration, nursing education, and policy making. For practice development, this study confirms the need to implement new working methods suggested by the nurses. This could improve adapting new working methods to clinical practice [65]. In regards to administration, nurses need administrative support to develop practice. Administrators should make sure that there are targeted individuals behind the decision making, in addition to evidence-based knowledge. When it comes to training, more comprehensive observation skills related to interpreting signs or triggers of violence are needed. Some nurses have a natural ability for engaging with patients. However, as a nurse’s behavior can also provoke patients in a negative sense, most nurses would need training for these events. Information delivered to patients about possible high-risk situations should be part of care, both at the beginning and during treatment. Otherwise, such situations may be provocative simply by coming up suddenly. In regards to policy making in psychiatric nursing, new tools are needed to relieve the overburdening of wards and climates of cynicism. De-escalation techniques, taught as policy in Finland since the 1990’s, have not resulted in clear change. De-escalation training programs should focus less on skills of physical restraint and more on competent interactions respecting patients’ perspectives [66].
